# False aneurysm of the interosseous artery and anterior interosseous syndrome - an unusual complication of penetrating injury of the forearm: a case report

**DOI:** 10.1186/1749-799X-4-44

**Published:** 2009-12-24

**Authors:** Ramon Pini, Stefano Lucchina, Guido Garavaglia, Cesare Fusetti

**Affiliations:** 1 Hand Surgery Unit, Ospedale San Giovanni, Bellinzona, Switzerland

## Abstract

**Background:**

Palsies involving the anterior interosseous nerve (AIN) comprise less than 1% of all upper extremity nerve palsies.

**Objectives:**

This case highlights the potential vascular and neurological hazards of minimal penetrating injury of the proximal forearm and emphasizes the phenomenon of delayed presentation of vascular injuries following seemingly obscure penetrating wounds.

**Case Report:**

We report a case of a 22-year-old male admitted for a minimal penetrating trauma of the proximal forearm that, some days later, developed an anterior interosseous syndrome. A Duplex study performed immediately after the trauma was normal. Further radiologic investigations i.e. a computer-tomographic-angiography (CTA) revealed a false aneurysm of the proximal portion of the interosseous artery (IA). Endovascular management was proposed but a spontaneous rupture dictated surgical revision with simple excision. Complete neurological recovery was documented at 4 months postoperatively.

**Conclusions/Summary:**

After every penetrating injury of the proximal forearm we propose routinely a detailed neurological and vascular status and a CTA if Duplex evaluation is negative.

## Introduction

Penetrating isolated lesions of the interosseous anterior neurovascular bundle are rare. We report the case of a 22-year-old male who sustained such a lesion with formation of a false aneurysm of the proximal portion of the interosseous artery (IA). A review of the literature showed one similar case of infective origin so that our description is the first of post-traumatic vascular compression of the anterior interosseous nerve (AIN) [1].

## Case Report

**Figure 1:**
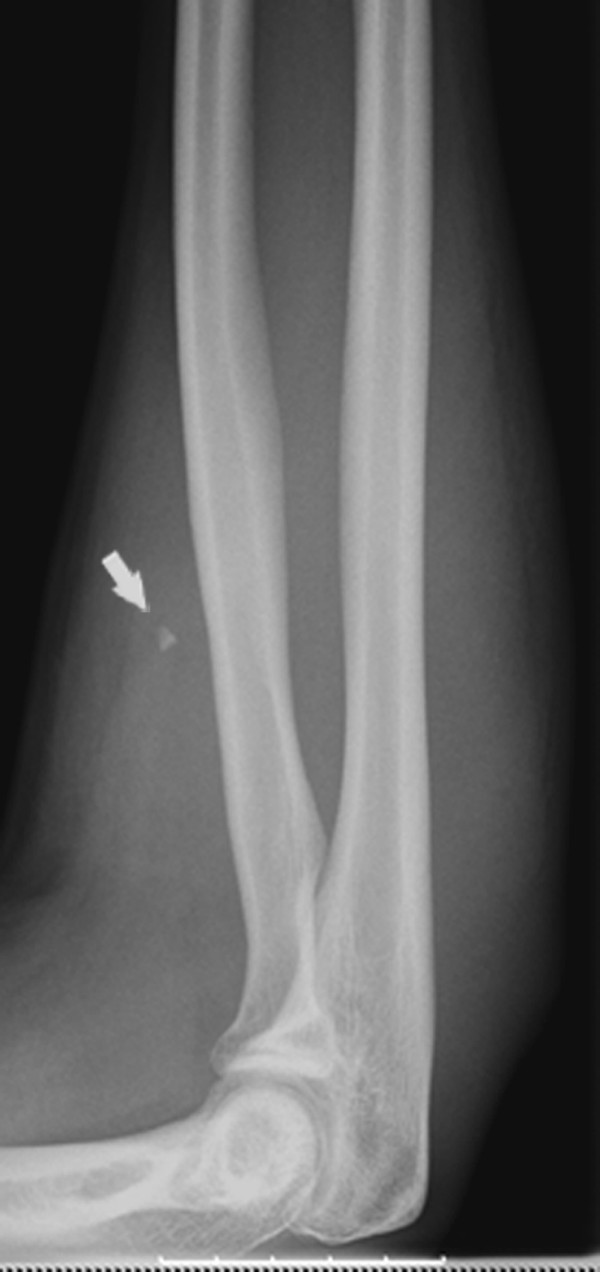
**X-ray**. The arrow shows the glass-like foreign body in the forearm.

**Figure 2:**
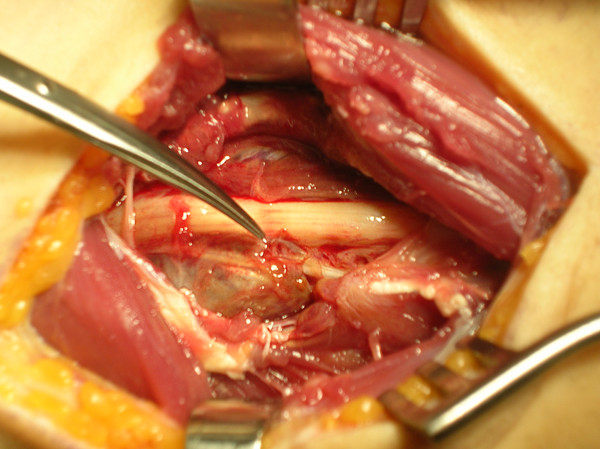
**Intraoperative picture**. The scissors shows the glass-like fragment lodged exactly into the first motor bifurcation of the anterior interosseous nerve (AIN). The main nerve is undamaged.

A 22 year old male sustained a penetrating injury of the forearm, after falling into a glass window during his stay in the Far East. The initial haemorrhage was treated with a simple compression. X-ray showed a small glass-like foreign body (fig. 1). A Duplex study was apparently normal. A few days later, he developed a rapidly complete sensory deficit on the median nerve and a loss of motor function on the AIN. No specific therapy or further investigation was proposed to the patient, who, back home 4 weeks later, consulted our unit. An established ischemic contracture (Holden moderate type) was clinically suspected. Electrophysiological studies confirmed the neurological lesion, with partial denervation of the flexor pollicis longus (FPL), coupled with moderate reduction of sensitive conduction in the median and ulnar nerves. We decided on surgical exploration. An extensive hematoma in the flexor's compartment was drained with extraction of the glass fragment which was lodged exactly in the first motor bifurcation of the AIN. The main trunk of the AIN was undamaged (fig. 2). The motor branch was reconstructed with a nerve graft. In the absence of evidence of a vascular lesion and active bleeding, a simple fasciotomy was performed before skin closure.

**Figure 3:**
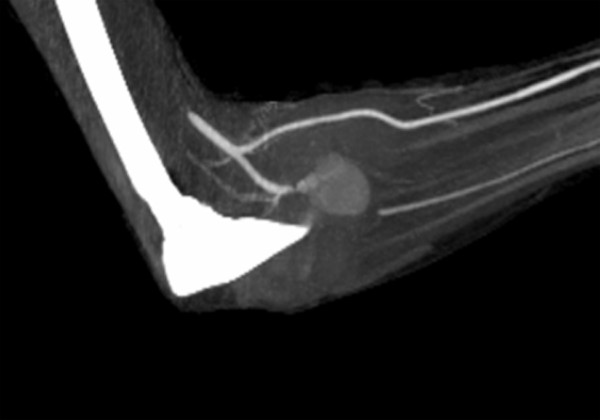
**Angio-CT-Scan**. False aneurysm of the proximal portion of the interosseous artery (IA).

Two days later the arm became newly swollen and painful. A computer-tomographic-angiography (CTA) (fig. 3) confirmed a false aneurysm of the IA. An endovascular embolisation was planned, but suddenly excruciating pain dictated an immediate surgical revision with aneurysm excision and arterial ligation. A complete neurological recovery was documented four months later.

## Discussion

Upper extremity injuries constitute 30-50% of all peripheral vascular injuries, more than 80% of which are from penetrating trauma. Radial and ulnar arterial injuries make up 5-30% of all peripheral vascular injuries [2]. The most common cause of upper extremity vascular injuries is penetrating trauma secondary to gunshot wounds, stab wounds and lacerations from broken glass. However, iatrogenic traumas secondary to the widespread use of diagnostic and therapeutic intravascular techniques have also contributed to the increase in incidence [2,3].

Nerve compression from a false aneurysm is extremely rare. A review of the literature showed one similar case of infective origin and two other cases with compression of the posterior interosseous nerve [1,4,5]. All the other cases in the arm were related to compression of the brachial plexus, median and ulnar nerve [6-13]. The peculiarity of our case report is the neurologic involvement of the AIN.

The AIN branches from the median nerve between the two heads of the pronator teres muscle, just distal to the origins of the motor branches to the superficial forearm flexor muscles and then runs with the IA on the anterior surface of the interosseous membrane, between and deep into the FPL and flexor digitorum profundus 1+2, which it supplies [14].

A focused history and thorough physical examination, combined with a working knowledge of normal vascular anatomy, can help identify most vascular abnormalities of the upper extremity. Technologic improvements, such as Duplex and CTA, now allow accurate diagnosis by non-invasive methods [15,16]. Nevertheless, our case shows that in a perforating trauma of the arm a careful neurological examination must always be performed, otherwise minor neurological signs, i.e. FPL dysfunction could pass unobserved. Although conventional teaching usually holds that an electro-diagnostic study should not be done until about 3 weeks after the injury, in fact a great deal of important information can be obtained by studies carried out within the first week [17].

For the vascular status, the first additional diagnostic modality is the Duplex: less expensive, rapidly carried out and successful in detecting significant lesions such as false aneurysms, arteriovenous fistulae, and major vessel occlusions [18]. In case of negative or uncertain result, a CTA or a simple arteriography should be routinely performed [1,19,20].

Our preoperative diagnosis was a chronic Volkmann's contracture (Holden moderate type) possibly combined with a lesion of the AIN. During wound exploration with a pneumatic tourniquet (250 mmHg) we found a muscular laceration, an organized hematoma and a lesion of the first motor branch of the AIN. Our preoperative diagnosis was confirmed and the neurological deficit was attributed to compression from the hematoma. This is why the vascular bundle was not explored and the possibility of an arterial false aneurysm was at first not even considered.

Once the circulation had been restored (pneumatic tourniquet off) and the hematoma removed, the aneurysm had the possibility to re-expand. This explains why the lesion became clinically and radiologically evident postoperatively.

Technically we used a proximal anterior approach. The dissection was difficult due to the old hematoma. We do not have a clear explanation for the acute bleeding during the night but do not think that it was due to an iatrogenic lesion during the first operation.

With regard to the pathogenesis, we assume that the false aneurysm is the result of a partial laceration of the IA due to the glass fragment found in the first motor bifurcation of the AIN. We were not able to visualize the path of the glass fragment because of the hematoma and the time lapse between the accident and the exploration. Retrospectively, the partial lesion of the IA by the glass fragment with secondary formation of a false aneurysm can explain both the hematoma and the anterior interosseous syndrome. The glass fragment in the first motor bifurcation cannot be the sole explanation of the anterior interosseous syndrome because the main trunk was intact. The compression of the AIN from the false aneurysm is, however, instead a plausible explanation.

For the secondary revision we believe that an endovascular approach would have been appropriate, to avoid a new dissection after the first microsurgical suture. Likewise, a primary endovascular approach could be considered, but only after a correct preoperative diagnosis and only in the absence of an extrinsic hematoma as the compression on the nerve would remain.

However, because it is a rare condition, this kind of approach has not been described in the literature and in our case an unexpected rupture of the aneurysm during the night imposed the classical and simple intervention with resection of the aneurysm. Similar to the cases described by Illuminati and Kim the results were optimal [1,21].

Nowadays, nonsurgical approaches play an important role in the treatment of peripheral false aneurysms [16,22]. Endoluminal repair of false aneurysms, large arteriovenous fistulas, intimal flaps, and focal lacerations, is performed by using stent-graft technology. Castelli and colleagues reported a 100% immediate success rate in managing axillo-subclavian arterial injuries [23]. In the study of Onal *et al*, all the stent-grafts in 17 patients with iatrogenic, traumatic, or spontaneous vascular lesions, were deployed successfully [24]. However, careful patient selection must be emphasized [25]. In the study of Komorowska-Timek *et al*, the ultrasound-guided thrombin injection has been shown to be effective also in the treatment of peripheral pseudo-aneurysms of the radial and ulnar artery [26].

## Conclusions

Penetrating injuries of the proximal forearm should not be underestimated because they are a potential cause of nerve and vascular injury.

While lesions of the major neuro-vascular bundels are often evident at clinical examination, this might not be the case for less accessible structures such as the anterior or posterior interosseous neurovascular bundle. The consequences can be disastrous, with development of sub-acute compartment syndrome or delayed diagnosis of neuro-muscular deficits, that have a less favourable functional prognosis.

We believe that for every penetrating injury of the forearm, the emergency physician should perform a detailed neurological and clinical status, evaluating not only sensibility but also every muscle group. Vascular examination distal to the lesion is mandatory, although with deep vascular lesions it might initially appear to be normal. As for clinical examination, Doppler investigation distal to the lesion is often seem to be normal, initially. We therefore recommend carrying out a detailed clinical and neurological status in association with a Duplex examination of the injured region, and if there is doubt, a CTA.

## Consent

Written informed consent was obtained from the patient for publication of this case report and accompanying images.

## Competing interests

The authors declare that they have no competing interests.

## Authors' contributions

RP has made substantial contributions to conception and design, or acquisition of data. RP, SL, GG, CF have been involved in drafting the manuscript or revising it critically for important intellectual content. RP, SL, GG, CF have given final approval of the version to be published.
